# *Plasmodium knowlesi* Genome Sequences from Clinical Isolates Reveal Extensive Genomic Dimorphism

**DOI:** 10.1371/journal.pone.0121303

**Published:** 2015-04-01

**Authors:** Miguel M. Pinheiro, Md Atique Ahmed, Scott B. Millar, Theo Sanderson, Thomas D. Otto, Woon Chan Lu, Sanjeev Krishna, Julian C. Rayner, Janet Cox-Singh

**Affiliations:** 1 School of Medicine, University of St Andrews, Medical and Biological Sciences Building, North Haugh, St Andrews, United Kingdom; 2 Malaria Research Centre, University Malaysia Sarawak, Kuching, Sarawak, Malaysia; 3 Malaria Programme, Wellcome Trust Sanger Institute, Wellcome Trust Genome Campus, Hinxton, Cambridge, United Kingdom; 4 Sarikei Hospital, Sarikei, Sarawak, 96100, Malaysia; 5 Division of Clinical Sciences, St. George’s, University of London, London, United Kingdom; Institute of Tropical Medicine, Nagasaki University, JAPAN

## Abstract

*Plasmodium knowlesi* is a newly described zoonosis that causes malaria in the human population that can be severe and fatal. The study of *P*. *knowlesi* parasites from human clinical isolates is relatively new and, in order to obtain maximum information from patient sample collections, we explored the possibility of generating *P*. *knowlesi* genome sequences from archived clinical isolates. Our patient sample collection consisted of frozen whole blood samples that contained excessive human DNA contamination and, in that form, were not suitable for parasite genome sequencing. We developed a method to reduce the amount of human DNA in the thawed blood samples in preparation for high throughput parasite genome sequencing using Illumina HiSeq and MiSeq sequencing platforms. Seven of fifteen samples processed had sufficiently pure *P*. *knowlesi* DNA for whole genome sequencing. The reads were mapped to the *P*. *knowlesi* H strain reference genome and an average mapping of 90% was obtained. Genes with low coverage were removed leaving 4623 genes for subsequent analyses. Previously we identified a DNA sequence dimorphism on a small fragment of the *P*. *knowlesi normocyte binding protein xa* gene on chromosome 14. We used the genome data to assemble full-length *Pknbpxa* sequences and discovered that the dimorphism extended along the gene. An in-house algorithm was developed to detect SNP sites co-associating with the dimorphism. More than half of the *P*. *knowlesi* genome was dimorphic, involving genes on all chromosomes and suggesting that two distinct types of *P*. *knowlesi* infect the human population in Sarawak, Malaysian Borneo. We use *P*. *knowlesi* clinical samples to demonstrate that *Plasmodium* DNA from archived patient samples can produce high quality genome data. We show that analyses, of even small numbers of difficult clinical malaria isolates, can generate comprehensive genomic information that will improve our understanding of malaria parasite diversity and pathobiology.

## Introduction

*Plasmodium knowlesi* is a malaria parasite of old world macaques that causes zoonotic malaria in humans [1]. *P*. *knowlesi* has been widely used as an experimental model leading to seminal discoveries in aspects of malaria biology, including antigenic variation, vaccine development and erythrocyte invasion (for example [2,3,4]). More recently, the discovery of severe cases of *P*. *knowlesi* malaria in the human population has re-kindled human-disease focussed research on this important parasite [5]. *P*. *knowlesi* lacks unique morphological characteristics and human infections are often mis-diagnosed as *P*. *malariae* or other *Plasmodium* species[6]. Novel *P*. *knowlesi*-specific PCR assays now allow accurate identification of *P*. *knowlesi* malaria and PCR-confirmed cases are continuously reported across Southeast Asia, including severe and fatal cases in Malaysia [7,8,9,10].

*P*. *knowlesi* is a widespread human infectious agent in Southeast Asia, yet we currently know very little about naturally circulating parasite populations that enter the human host or the factors that are associated with severe disease. In Sarawak, Malaysian Borneo, we found that *P*. *knowlesi* parasitaemia is associated with disease severity [8,9]. To study the relationship between parasitaemia and variation in the proteins that are involved in invasion of human erythrocytes, short regions of two *P*. *knowlesi* invasion genes, *P*. *knowlesi normocyte binding protein (Pknbp) xa* and *Pknbpxb*, were sequenced from more than 100 human infections [11]. Both gene fragments were polymorphic and the *Pknbpxa* fragment was dimorphic with distinct co-associating polymorphisms that segregated into two clusters. In the study cohort, patients were infected with parasites with either *Pknbpxa* dimorphic type at almost equal frequency but only alleles found in one dimorphic type associated with markers of disease severity [11]. While this suggests a potential link between invasion phenotypes, parasitaemia and virulence, it is critical to extend the study beyond a candidate gene level and out to the whole genome.

A reference *P*. *knowlesi* genome sequence has been generated from the macaque-adapted experimental H strain [12], but *P*. *knowlesi* genome sequences from clinically well-characterised isolates are not currently available. The generation of parasite genome sequences from clinical *Plasmodium* samples requires a leucocyte depletion step to minimise the amount of contaminating human DNA. However, many archived sample collections exist, including our own collection of frozen whole blood samples from patients with *P*. *knowlesi* malaria, that have not been leucocyte depleted before freezing. Adapting depletion approaches to these frozen sample sets would unlock a wealth of genomic information.

Here we report a method to deplete human DNA from frozen clinical malaria samples and render them suitable for whole genome sequencing. The method exploits two assumptions; 1) that not all leucocytes are lysed when whole blood goes through one freeze/thaw cycle and 2) the more robust parasites would survive the same treatment either in intact infected red blood cells (IRBCs) or as free parasites released from lysed erythrocytes. We developed a simple filtration method to remove leucocytes and recover parasite-rich pellets for *Plasmodium* genome sequencing. The method offers the malaria research community a means to interrogate *Plasmodium* species genome data in important archived sample collections. In this case, we use the approach to generate genome sequence data from six previously frozen *P*. *knowlesi* clinical isolates, and show that the *Pknbpxa* dimorphism may extend across the *P*. *knowlesi* genome.

## Materials and Methods

### Patient samples

Archived frozen whole blood samples were used from adult patients recruited into a non-interventional study with informed signed consent that included use of samples in related studies. Patient consent forms are securely stored in the University of St Andrews. Patient recruitment and consent protocols were approved by the Medical Research and Ethics Committee, Ministry of Health Malaysia and the Ethics Committee Faculty of Medicine and Health Sciences, University Malaysia Sarawak. The use of the samples in the study reported here was further approved by the University of St Andrews Teaching and Research Ethics Committee.

### Human DNA depletion using Whatman filter paper

EDTA blood samples from *P*. *knowlesi* patients were collected and stored at -40°C. The samples were thawed and the volume measured before gentle mixing in ice-cold PBS at a ratio of 300ul thawed blood per 5ml cold PBS. The mixture was pipetted into a 10mL syringe barrel, the base was lined with 3 layers of Whatman No 3 (6uM pore size) to remove small lymphocytes and 3 layers of Whatman No 1 (11uM pore size) on top to remove larger surviving leucocytes. The filter papers were cut to fit the internal diameter of the syringe and pre-wet with PBS before use. Not more than 10mL of diluted blood was loaded per syringe column. The filtrate was collected into sterile 50mL centrifuge tubes following centrifugation at 125g for 2 minutes at 4°C. The columns were washed through with 10mL volumes of cold PBS and each wash was collected into the filtrate tube by centrifugation as above until the filters were no longer blood-stained. The total combined filtrate, up to 40 mL, was centrifuged at 2000g for 10 minutes at 4°C to pellet any surviving IRBCs and free parasites. Pellets were re-suspended in 1ml cold PBS and transferred to 1.5ml Eppendorf tubes and recovered by centrifugation at 14,000g, for 2 minutes at 4°C. Pellets were re-suspended and washed in 1mL cold PBS and collected by centrifugation as described. This wash step was repeated two more times. The washed IRBC/parasite pellets were suspended in 20ul Proteinase K (QIAGEN) followed by 200ul cold PBS. The mixture was vortexed thoroughly before DNA extraction using QIAamp Blood Mini kit (QIAGEN) with RNase A, as per manufacturers instructions. For samples with more than 100,000 parasites /ul blood the initial blood dilution step was 150ul thawed blood into 5mL cold PBS.

### TaqMan qPCR multiplexed for human and *P*. *knowlesi* DNA

*Plasmodium* specific 18ssURNA Plasmo1: 5′ GTTAAGGGAGTGAAGACGA TCAGA and Plasmo 2: 5′ AACCCAAAGACTTTGATTTC TCATAA primers were used [13] with the published *P*. *knowlesi* TaqMan probe 5′ CTCTCCGGAGATTAGAACTCTTAGATTGCT labelled with 5'FAM and 3'BHQ1 [14]. Human DNA primers: Plat1-A 5′ CTTACCACATCCGCTCCATC, and Plat1-B 5′ TTCACACTCTCCGTCACATTG with the probe 5′ HEX/CACATCCCC/ZEN/AGTGCCGAGTTAGA/3IABkFQ were used. The qPCR master mix contained 250nM Plasmo1, 250nM Plasmo2, 250nM Plat1-A, 250nM Plat1-B, 125nM *Pk* probe, 125nM Plat1-Probe, 1 x Roche RT-PCR Master Mix and 1ul DNA template in 20ul final volume. qPCR cycling was 10 minutes at 95°C, followed by 45 cycles of 10 seconds at 95°C, 30 seconds at 57°C, and 1 second at 72°C using the Roche LightCycler 480 II.

### Illumina sequencing

DNA was quantified (Qubit Fluorometric Quantitation, Invitrogen, Life Technologies) and sheared into fragments of 400–600 bp. Illumina libraries were generated using a) the PCR free protocol (NoPCR) [15] or b) the standard library preparation using the KAPA enzyme [16] with eight PCR cycles. NoPCR libraries were sequenced on the Illumina HiSeq 2000 platform for 100 paired-end cycles and standard PCR libraries were sequenced on Illumina MiSeq for 150 paired ends cycles using V4 or V5 SBS sequencing kits and proprietary reagents according to manufacturer's recommended protocol (https://icom.illumina.com/). Data were analysed from the Illumina sequencing machines using RTA1.6, RTA1.8 or GA v0.3 analysis pipelines.

### Reference genome

The *Plasmodium knowlesi* H strain reference genome version 11.1 GeneDB (www.genedb.org/Homepage/Pknowlesi) was downloaded from PlasmoDB (www.plasmodb.org) [12,17,18]. The region corresponding to the *pknbpxa* gene (PKH_146970 and PKH_146980) in chromosome 14 was partially missing and fragmented in the current reference genome and we corrected for this using the published *pknbpxa* gene sequence (GenBank accession number EU867791.1) [2]. Common non-coding DNA regions upstream and downstream of the *pknbpxa* gene were located in both the *Plasmodium knowlesi* strain H reference genome and the published *pknbpxa* gene. With this information it was possible to replace the *pknbpxa* gene (PKH_146970) in the reference genome sequence with the published EU867791.1 gene sequence without disrupting subsequent mapping. The *pknbpxb* gene, which was not annotated correctly, was rectified using the EU867792.1 published gene sequence [2].

### Genome sequence mapping

HiSeq and MiSeq reads from *P*. *knowlesi* enriched, human DNA depleted, samples are deposited in the EMBL-EBI European Nucleotide Archive (http://www.ebi.ac.uk)[19]. The archive references are for HiSeq: ERR274221; ERR274222; ERR274224; ERR274225 and MiSeq: ERR366425 and ERR366426. Sequences mapping to the human genome, representing patient DNA, were removed from this data in the sequencing pipeline. The reads were mapped to the corrected *P*. *knowlesi H* strain reference genome sequence (PlasmoDB-11.1_PknowlesiH_Genome.fasta) using Bowtie-2 [20] followed by Bedtools to summarise the coverage of each genome [21].

### Single Nucleotide Polymorphism (SNP) calling

Samtools mpileup with threshold base quality set to 13 was used with BCFtools to generate Variant Call SNP Format (VCF) files for each *P*. *knowlesi* genome sequence [22]. A varFilter (BCFtools) was applied and all SNP sites with allele frequency less than 0.9 were removed. Insertions and deletions were not included in any of the analyses or scripts. Only SNP sites with a minimum coverage of 13 were taken into consideration.

### Linkage Disequilibrium analysis of full-length *pknbpxa* sequences extracted from *P*. *knowlesi* genome sequence data

We used Artemis [12,17,23,24] and the VCF files to generate full-length *pknbpxa* gene sequences as fasta files from each of the genome sequences (n = 6). The fasta files were converted to the Haploview compatible PLINK format [25]. Linkage disequilibrium was performed on the full-length coding region of *Pknbpxa* sequences using Haploview and analysed using default parameters [26,27]. Nucleotide diversity (π) was calculated using a 400bp window length with a step size of 25bp, DnaSP [28].

### Identification of polymorphisms genome-wide co-associating with the *Pknbpxa* fragment dimorphism

**Fig 1:**
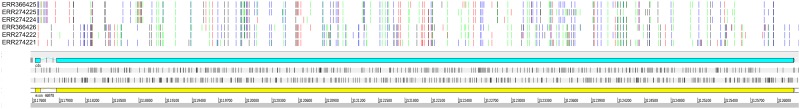
A screen shot of Artemis DNA view comparing six *Plasmodium knowlesi* genome sequences from patient isolates to the *Plasmodium knowlesi* H strain reference genome sequence. The *P*. *knowlesi normocyte binding protein xa* locus on chromosome 14 is shown. The screen shot shows segregation of the sequences from patient isolates into two groups, (n = 3 in each group) and the dimorphism is clearly visible.

An algorithm was developed to identify SNP sites in each genome sequence (n = 6), co-associating with the *P*. *knowlesi Pknbpxa* dimorphic pattern already identified in a small fragment of this gene [11] and also visible in Artemis on chromosome 14 at the *Pknbpxa* locus (Fig. 1). Briefly the script was designed to screen VCF files to identify each SNP and test if the SNP co-associated with SNPs defining the *Pknbpxa* dimorphism. Co-associating patterns were predefined in the algorithm to describe which kind of symbols (SNP pattern) each genome required to fit within either of the *P*. *knowlesi Pknbpxa* dimorphic forms. Every time a SNP fit the pattern the event was signalled (recorded). Finally, an image was created to show the density of all SNP sites and then the co-associating SNPs for each chromosome (S1 Fig.).

### Testing the distribution of co-associated SNPs defined in the *Pknbpxa* dimorphism

To identify positions on each *P*. *knowlesi* chromosome where the density of co-associating sites was more evident a Chi square test of independence was applied followed by a calculation of adjusted residuals. For this, each chromosome was divided into 30 equal parts and a contingency table was created to reflect the number of SNPs co-associating with the *Pknbpxa* dimorphism per part per chromosome. Adjusted residuals were calculated in a contingency table and a threshold of > 3.00 for more co-associating SNPs than expected and < -3.00 for less co-associating dimorphic SNPs than expected was applied to the resulting values. By applying these thresholds it was possible to identify, within 99.7% limits of confidence, co-associating SNP sites for each chromosome with higher or lower than expected co-associating SNP density.

### Gene Ontology (GO)

*P*. *knowlesi* genes were analysed using Blast2GO http://www.blast2go.com version 2.7.2 [29]. All genes with complete coverage (4623) were blasted against nr@ncbi database and an InterProScan 5 analysis was performed [30]. The Gene Ontology classification was done with default parameters [31]. Genes with no (0) co-associating SNP sites, with >0–<10 (1–9) and >9 (≥ 10) SNPs that co-associated with the *P*. *knowlesi* genome-wide dimorphism were identified within each resulting GO group.

### Testing for enrichment of dimorphic genes in particular GO subgroups

Genes with dimorphic SNP sites were tested for statistically significant enrichment of dimorphic genes in GO subgroups using *topGO* Enrichment analysis for Gene Ontology. R package version 2.14.0. Adrian Alexa and Jorg Rahnenfuhrer (2010). (http://www.bioconductor.org/packages/release/bioc/html/topGO.html). For this we selected two groups of genes those with one or more dimorphic SNP sites (≥1) and a separate group with ten or more dimorphic SNP sites (≥10) and analysed for enrichment against all genes with at least one SNP whether or not dimorphic.

## Results

### Human DNA depletion from frozen whole blood samples

Frozen whole blood samples from fifteen *P*. *knowlesi* patients, with parasite counts ranging from 10,000–400,000 parasite/ul, were thawed and human DNA depleted using an in-house method. Briefly white blood cells were removed by filtration through Whatman filter paper followed by parasite recovery as described in detail (see Methods section). Total human and parasite DNA was quantified using qPCR (Table 1). Nine of fifteen isolates had the required >100ng of *P*. *knowlesi* DNA, and seven of the nine had <80% human DNA contamination, the cut-off for *Plasmodium* genome sequencing, and were suitable for sequencing (Table 1). Five and two DNA samples were used to generate NoPCR and PCR sequencing libraries and multiplexed in a single lane on Illumina HiSeq and MiSeq platforms respectively (Table 1). The remaining six samples had insufficient *P*. *knowlesi* DNA and/or >80% human DNA (hDNA) contamination (Table 1).

**Table 1:** Clinical samples human DNA depleted using the Whatman filtration method.

Sample ID	Parasites/ul	Accession ID^§^	Total hDNA (ng) qPCR	Total Pk DNA (ng) qPCR	% Pk DNA
47	128,500	ND	302	294	49.3
Duplicate		ERR366425*	131	238	64.5
87	47,000	NS	225	51	18.7
Duplicate		NS	6	8	56
73	40,000	ERR366426*	33	233	87.6
Duplicate		ND	106	162	60.4
91	29,000	NS	269	37	12
220	10,000	NS	735	24	3.1
48	86,000	ND	1402	1167	45.4
Duplicate		ERR274221**	421	824	65.7
50A	139,000	ERR274222**	317	2775	89.8
Duplicate		ND	415	3280	88.8
50B	390,000	ERR274223	597	1239	67.5
55	186,500	NS	1235	428	25.7
Duplicate		NS	3418	433	11.2
58	149,000	ERR274224**	336	506	60
62	139,000	NS	988	71	6.7
178	104,000	NS	103	11	9.4
233	326,000	NS	1015	313	23.5
258	58,000	NS	560	94	14.3
Duplicate		NS	245	59	19.4
299	66,000	ERR274225**	626	984	61.1

Samples sequenced using Illumina HiSeq or MiSeq sequencing platforms are labelled. The amount of Parasite DNA recovered and per cent human DNA contamination are given.

*MiSeq.

**HiSeq.

NS = Not suitable.

ND = Duplicate sample suitable but not sequenced.

^§^ Accession number EMBL-EBI European Nucleotide Archive (http://www.ebi.ac.uk).

### DNA obtained from frozen *P*. *knowlesi* clinical isolates generated high coverage genome sequence

*P*. *knowlesi* sequence data was generated from seven patient isolates, five from HiSeq runs and two from MiSeq runs. The HiSeq runs generated >36 million reads and MiSeq >5 million reads. An average mapping of >90% was obtained for both HiSeq and MiSeq data producing an average coverage of >140x for HiSeq and >30x for MiSeq. The total number of reads mapped and not mapped, percent human DNA contamination and coverage per genome sequence are summarized in Table 2.

**Table 2:** *P*. *knowlesi* clinical isolate genome sequence summary report.

Sequence ID	ERR366426	ERR366425	ERR274221	ERR274223	ERR274222	ERR274225	ERR274224
Technology	MiSeq	MiSeq	HiSeq	HiSeq	HiSeq	HiSeq	HiSeq
Sample Ref.	SKS-047	SKS-073	SKS-048	SKS-050B	SKS-050A	SKS-299	SKS-058
Total Reads	6003990	6130562	45217760	47211338	58756176	51769792	41469862
Total Reads Mapped	4731148	5049125	37095534	38967367	48466910	43203711	34547072
Total Reads Not Mapped	1272842	1081437	8122226	8243971	10289266	8566081	6922790
% Reads Not Mapped	21	17	17	17	17	16	16
% Human DNA	1.58	1.59	0.75	0.48	0.14	0.71	0.73
Coverage	32	34	166	175	218	195	156
Number of SNPs	267055	260888	318427	317051	317072	304641	304147
Number Dimorphic SNPs	42771	42771	42771	42771	42771	42771	42771
% Zero Coverage (%)	7.8	7.9	5.5	5.8	5.8	5.9	6
Coverage >1 (%)	91.6	91.4	94.1	93.9	93.9	93.7	93.6
Coverage >5 (%)	90.1	89.9	93.5	93.3	93.4	93.1	92.9
Coverage >10 (%)	88.4	88.4	92.9	92.8	93	92.6	92.4

Genome Size 23487363

### *P*. *knowlesi* genome analysis

The reads were mapped to the *P*. *knowlesi* H strain reference genome following correction of the *Pknbpxa* locus (see materials and methods section). Two genome sequences (ERR274222 and ERR274223) were generated from a single patient representing pre- and post-treatment samples. Only the pre-treatment sample sequence, ERR274222, was included in subsequent analyses along with sequences from five other patients all collected pre-treatment.

The sequences covered 5228 genes, including genes and gene fragments annotated as genes of un-known function. Data from 605 genes were excluded because coverage was zero at one or more base position leaving 4623 genes in subsequent analyses. This filter excluded all but five of the 195 SICAVar genes and gene fragments and all but three of the 67 KIR genes and gene fragments. Both of these gene families are highly polymorphic, and the gene sequences in these contemporary clinical isolates are likely to be very different to those in the historical monkey-adapted reference genome, so mapping issues and low coverage in these gene sets is to be expected. Of the remaining genes 2180 (47.2%) were annotated as genes with unknown function. The SNP distribution across the genome is shown in S1 Fig.

### Dimorphism extends across and beyond *Pknbpxa*

In a previous study we identified a DNA sequence dimorphism in a fragment (885bp) representing 10% of the *P*. *knowlesi* normocyte binding protein (*Pknbp)xa* gene that codes for a protein involved in red blood cell invasion. To determine the extent of the dimorphism across the gene, full-length *Pknbpxa*, (PKH_146970) coding sequences were assembled from the six genome sequences obtained from the same patient cohort. Ninety-one (91) *Pknbpxa* SNPs co-associated with the dimorphic pattern (r^2^ = 1). This dimorphism effectively divides the *Pknbpxa* gene sequences into two clusters of sequence types, with *Pknbpxa* sequences from three genomes falling into cluster 1 and three into cluster 2. Nucleotide diversity (π) was higher across the clusters (π = 0.01441), than it was within each cluster, (π = 0.00518 for cluster 1 (n = 3) and π = 0.00868 for cluster 2; n = 3 each). Cluster 1 was less diverse than cluster 2, consistent with *Pknbpxa* nucleotide diversity found in the previous study, but the significance of this difference cannot be estimated based on six sequences.

**Fig 2:**
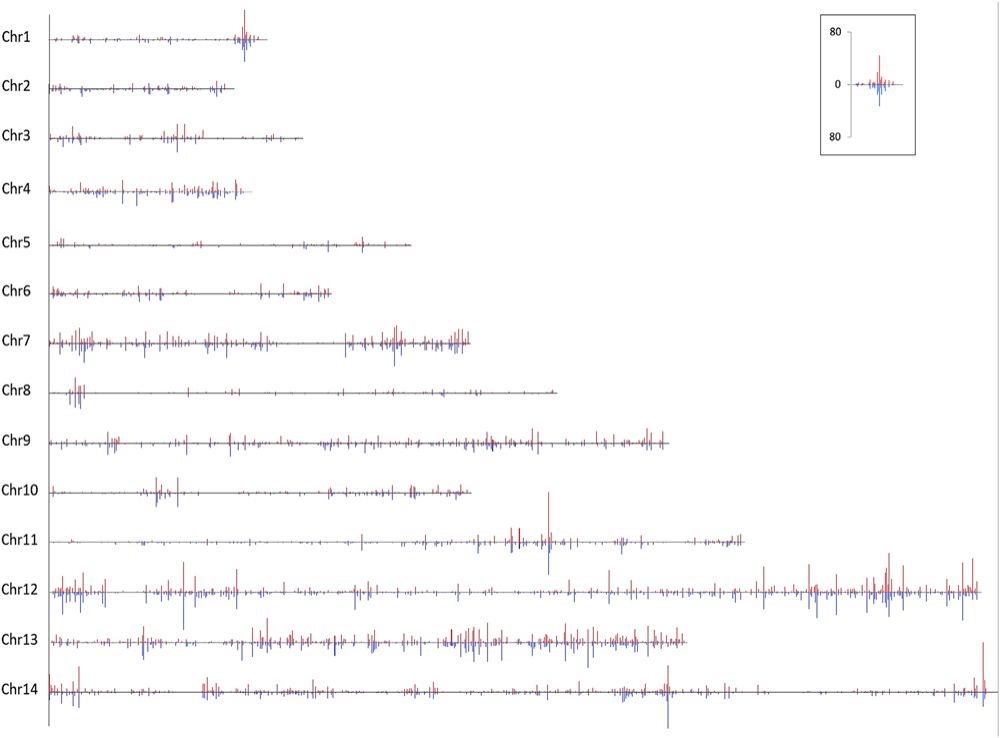
The number and position of SNP sites per gene co-associating with the *P*. *knowelsi* genome-wide dimorphism. Non-synonymous polymorphisms (red) are shown above the line and synonymous polymorphisms (blue) are shown below the line. The line is drawn at zero. The chromosomes are drawn to scale and the height of the bars represents the number of SNP sites per gene per region of each chromosome. The scale is given in the boxed area and is the number of SNP sites per gene.

The *P*. *knowlesi* genome sequences from clinical isolates were viewed in Artemis, a genome browser and annotation tool and referenced to the *P*. *knowesi* H strain genome sequence. Two SNP patterns emerged and the *Pknbpxa* dimorphism was clearly visible with sequences from three patient isolates clustering into each pattern (Fig. 1). To test whether the dimporphism extended beyond the boundaries of the *Pknbpxa* gene, SNP association with the *Pknbpxa* dimorphism was examined first along chromosome 14 and then genome-wide using an in-house script (see Materials and Methods section). The dimorphic SNP pattern was evident at multiple genetic loci on all chromosomes (S1 Fig.). The relative distribution of co-associated SNPs on each chromosome was determined by dividing each chromosome into 30 equal parts and using the Chi squared test of independence to test expected and observed events (S1 Table). Although the dimorphism extends across the full genome, the intensity and distribution is not uniform or clustered in any particular chromosomal region. The position and number of non-synonymous and synonymous SNPs co-associating with the dimorphism per gene per chromosome are represented in Fig. 2.

More than half of the *P*. *knowlesi* genes in the genome, 2801 of 4623 genes (60.8%), appear to be dimorphic. Within the dimorphic group the number of dimorphic SNP sites per gene varied widely. For example, while *Pknbpxa* had a total of 326 SNPs, of which 91 (27.9%) co-associated with the dimorphism, a related gene of similar size, *Pknbpxb*, had a total of 197 SNPs of which only 5 (2.2%) co-associated with the dimorphism (S2 Table). Applying a more conservative cut-off identified 507 genes with ≥10 co-associating SNPs, representing 11% of genes in the genome with adequate coverage. Of these 301 (59.5%) were annotated as genes of unknown function. The chromosome location and annotated function of the remaining 206 genes is listed in S2 Table. Notable genes within this high stringency dimorphic group included 12 of 27 (44%) of genes annotated as transcription factors with AP2 domains in the *P*. *knowlesi* genome. Several genes associated with drug resistance in other *Plasmodium* species, such as putative multi drug resistance-associated protein PkMRP1(PKH_144590) and putative multidrug resistance protein, PkMDR 2 (PKH_125840) S2 Table, were also dimorphic, while the putative chloroquine resistance transporter (CRT) gene (PKH_010710) had 23 SNPs, none were dimorphic and only one SNP conferred an amino acid change.

**Fig 3:**
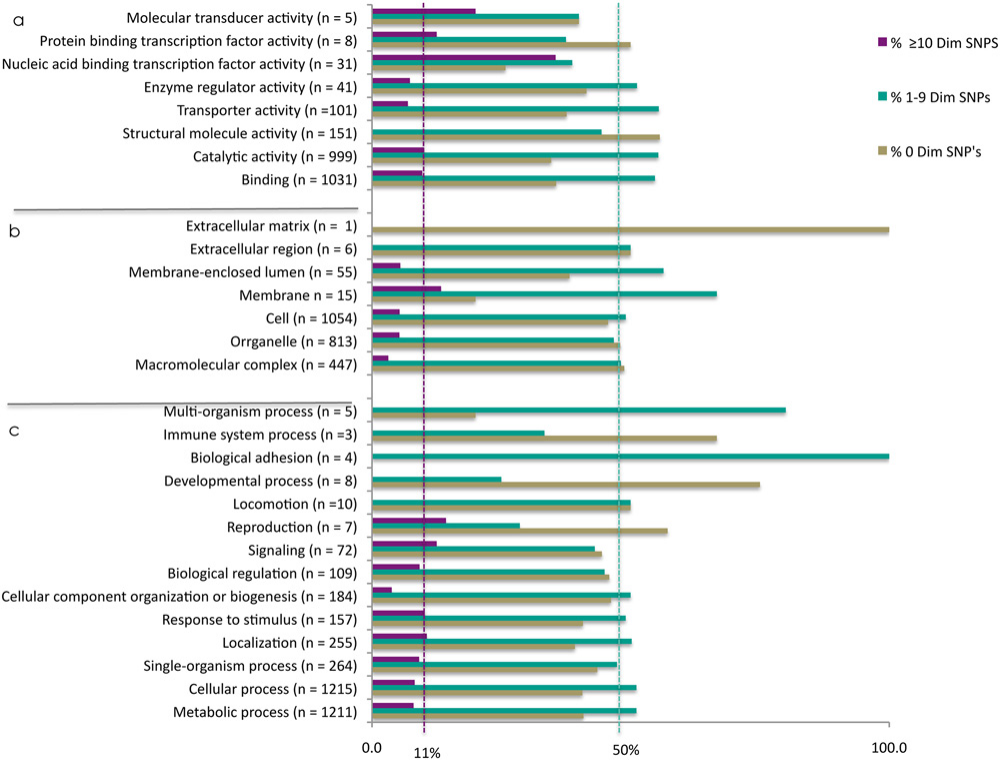
*P*. *knowlesi* genes are grouped by gene ontology (GO) terms. The percent of genes in each GO sub-group of a) molecular function, b) cellular processes and c) biological processes are shown, n = the total number of mapped annotated genes in each GO sub-group. Percent of genes in GO subgroups with: no dimorphic SNP sites shown in brown, genes with 1–9 dimorphic SNP sites turquoise and genes with ≥10 dimorphic SNP sites purple. The expected percent of genes with 1–9 dimorphic SNPs (50%) is marked with a turquoise hatched line and the expected percent of genes with ≥10 dimorphic SNPs (11%) is marked with a purple hatched line. Gene ontology was assigned using Blast2GO—Software for Biologists, http://www.blast2go.com.

The enrichment of dimorphic genes among genes encoding transcription factors with AP2 domains was obvious and identified manually. We then used Gene Ontology (GO), (Blast2GO) tools to examine whether other *P*. *knowlesi* dimorphic genes were enriched in GO groups that served particular biological functions. Genes were sorted into GO term groups and sub-groups with putative or known molecular function, cellular process activity and biological process activity (Table 3). We then calculated the proportion genes with ≥1 dimorphic SNP in each GO group (Table 3). Most of the GO term groups had, as expected, approximately 60% dimorphic genes but there was variation (Table 3). If dimorphic genes have evolved randomly over time then the proportion of genes with dimorphic SNP sites in the GO groups would not be expected to be different from the distribution of genes with dimorphic SNP sites in the whole genome that is: 39% of genes with no dimorphic SNPs; 50% of genes with 1–9 dimorphic SNPs and 11% of gene with ten or more dimorphic SNPs. Several GO sub-groups had more genes than expected with 1–9 dimorphic SNP sites and ≥10 dimorphic SNP sites for example genes with molecular transducer activity, nucleic acid binding transcription factor activity and membrane association (Fig. 3a, 3b and 3c). There were also sub-groups of genes where dimorphic SNP sites were under-represented, including structural and molecular activity, developmental process and immune system process (Table 3 and Fig. 3). We used *topGO* to test for statistically significant enrichment of dimorphic genes in GO term groups (Table 4). In the first instance all genes with at least one dimorphic SNP were analysed and there was significant enrichment, particularly in the ion binding function, helicase activity and tRNA metabolic process function GO term groups (Table 4). Genes with ≥10 dimorphic SNPs were significantly enriched in the nucleic acid binding transcription factor activity and kinase activity GO term groups (Table 4).

**Table 3:** Summary of *P*. *knowlesi* gene ontology (GO) analysis and the proportion of genes in each group with dimorphic SNP's.

Gene ontology (GO) group	GO-ID	GO- subgroup	Total number of genes	Total genes in dimorphism	Proportion in dimorphism
**Molecular function**	GO:0060089	Molecular transducer activity	5	3	0.60
	GO:0000988	Protein binding transcription factor activity	8	4	0.50
	GO:0001071	Nucleic acid binding transcription factor activity	31	23	0.74
	GO:0030234	Enzyme regulator activity	41	24	0.59
	GO:0005215	Transporter activity	101	63	0.62
	GO:0005198	Structural molecule activity	151	67	0.44
	GO:0003824	Catalytic activity	999	653	0.65
	GO:0005488	Binding	1031	664	0.64
**Cellular Processes**	GO:0031012	Extracellular matrix	1	0	0.00
	GO:0005576	Extracellular region	6	3	0.50
	GO:0031974	Membrane-enclosed lumen	55	34	0.62
	GO:0016020	Membrane	15	12	0.80
	GO:0005623	Cell	1054	573	0.54
	GO:0043226	Organelle	813	423	0.52
	GO:0032991	Macromolecular complex	447	229	0.51
**Biological processes**	GO:0051704	Multi-organism process	5	4	0.80
	GO:0002376	Immune system process	3	1	0.33
	GO:0022610	Biological adhesion	4	4	1.00
	GO:0032502	Developmental process	8	2	0.25
	GO:0040011	Locomotion	10	5	0.50
	GO:0000003	Reproduction	7	3	0.43
	GO:0023052	Signaling	72	40	0.56
	GO:0065007	Biological regulation	109	59	0.54
	GO:0071840	Cellular component organization or biogenesis	184	99	0.54
	GO:0050896	Response to stimulus	157	93	0.59
	GO:0051179	Localization	255	155	0.61
	GO:0044699	Single-organism process	264	149	0.56
	GO:0009987	Cellular process	1215	721	0.59
	GO:0008152	Metabolic process	1211	716	0.59

Gene Ontology assigned using Blast2GO—Software for Biologists, http://www.blast2go.com.

**Table 4:** *topGO* gene enrichment analysis.

Gene Ontology (Blast@GO)	*topGO* term functional description	*topGO* ID	≥1 dimorphic SNPs	≥10 dimorphic SNPs
**Molecular Function**			*p* =	*p* =
Binding	Ion binding	GO:0043167	0.000077	0.01011
Catalytic activity	Helicase activity	GO:0004386	0.000095	
Catalytic activity	ATPase activity	GO:0016887	0.0014	0.02964
Catalytic activity	Hydrolase activity, acting on glycosyl bonds	GO:0016798	0.0328	
Catalytic activity	DNA binding	GO:0003677	0.0121	
Catalytic activity	GTPase activity	GO:0003924	0.0421	
Nucleic acid binding transcription factor activity	Nucleic acid binding transcription factor activity	GO:0001071		0.000037
Catalytic activity	Kinase activity	GO:0016301		0.00067
**Cellular Component**				
Cell; Organelle	Microtubule organizing center	GO:0005815	0.021	
Cell	Plasma membrane	GO:0005886	0.037	
Cell; Organelle	Nucleus	GO:0005634		0.0029
Cell; Organelle	Cytoskeleton	GO:0005856		0.0402
**Biological Process**				
Metaboloc process; Cellular process	tRNA metabolic process	GO:0006399	0.00033	
Metaboloc process; Cellular process	Cellular amino acid metabolic process	GO:0006520	0.00357	
Single-organism process; Metaboloc process; Cellular process	Mitosis	GO:0007067	0.00849	
Metaboloc process; cellular process	Nucleobase-containing compound catabolic process	GO:0034655	0.01453	
Multi-organism process	Transport	GO:0006810	0.03512	
Metaboloc process; cellular process	Cellular protein modification process	GO:0006464	0.03678	
Metaboloc process; cellular process	Cellular nitrogen compound metabolic process	GO:0034641		0.028

## Discussion

Here we describe a method for enriching *Plasmodium* DNA from frozen whole blood samples collected from patients with malaria. The method required at least 200ul of whole blood at >40,000 parasites/ul to obtain sufficient parasite DNA for genome sequencing platforms. Parasite DNA recovery was inconsistent and human DNA contamination was the main problem. Nonetheless, seven of fifteen patient samples had sufficiently enriched *P*. *knowlesi* DNA to produce high quality genome sequences using Illumina sequencing platforms. The success may in part be because *P*. *knowlesi* is less AT rich (62%) than other *Plasmodium* genomes [12] perhaps reducing amplification bias. Combining the frozen sample filtration method described here with methylated DNA digestion and target enriched sequencing approaches described by others [16,32], may yield valuable *Plasmodium* genome data from many precious pre-existing frozen clinical sample collections.

In a previous study we identified a sequence dimorphism in a fragment (885bp) of the *P*. *knowlesi* normocyte binding protein (*Pknbp)xa* that codes for a protein involved in red blood cell invasion [11]. *Pknbpxa* dimorphic cluster 2 contained alleles associated with markers of disease severity implying that dimorphic cluster 2 may contain more virulent parasites than cluster 1. Our genome data revealed that the dimorphism extended along the full-length (>8000bp) *Pknbpxa* coding region, along chromosome 14 and beyond. SNPs co-associating with the *Pknbpxa* dimorphism were distributed genome-wide across all chromosomes.

Interestingly, even within the limitation that only six samples were sequenced, the dimorphism comprised numerous non-synonymous substitutions, suggesting, for the first time, that there may be at least two distinct types of *P*. *knowlesi* circulating in Sarawak, Malaysian Borneo, and that some may be more virulent that others. Dimorphic loci have been described in many *Plasmodium* species, particularly in merozoite surface antigens and invasion ligands of *P*. *falciparum* and *P*. *vivax* [33,34,35]. In *P*. *ovale* dimorphic characteristics at selected loci prompted the division of *P*. *ovale* into two sub-species [35]. Even so the evolution and maintenance of allelic dimorphisms in *Plasmodium* species is difficult to explain [34]. Here we demonstrate a genome-wide dimorphism, involving more than half of the genes in the *P*. *knowlesi* genome, including genes coding for functions that transcend from exposed parasite surfaces to protected internal sites. The sub-division of *P*. *knowlesi* into distinct types will require further sequence confirmation, yet the genome-wide nature of the dimorphism is striking.

Although there was significant enrichment of dimorphic genes in several GO functional groups it is not clear what is driving a genome-wide dimorphism in *P*. *knowlesi*. Interestingly twelve genes implicated in parasite lifecycle stage-specific transcription, the putative transcription factors with Apicomplexan Apetala2 (AP2) domains [36,37,38,39] were dimorphic. Variation at these loci may mark genetically distinct lifecycle characteristics isolating *P*. *knowlesi* into strains or subspecies. In addition, all nine members of the ABC, ABC C transporter protein family of genes, annotated in the *P*. *knowlesi* genome, were dimorphic [12,40]. These genes are found in all phyla and represent an ancient gene family that, in eukaryotes, expel a wide range of unwanted substrates [41]. This family of genes include *P*. *knowlesi PkMDR2* and *PkMRP1* that were both polymorphic and dimorphic implying selection pressure at these loci. *PkMDR2* and *PkMRP1* are orthologues of *P*. *falciparum PfMDR 2* and *PfMRP1*, genes that carry genetic markers of drug resistance, including resistance to mefloquine [40,42]. Tantalizingly, experimental lines of *P*. *knowlesi* were found innately resistant to mefloquine in Rhesus monkeys and clinical isolates did not respond well to mefloquine *ex vivo* [43,44]. Patients with uncomplicated *P*. *knowlesi* infections responded to mefloquine but one patient with severe disease exhibited RIII type resistance [45,46,47].

Selection at these promiscuous transporter loci in zoonotic parasites that, unlike *P*. *falciparum*, are not under conventional drug selection pressure may at first seem surprising. However, domestic and wild animals eat plants with bio-active properties—they self-medicate [48]. The jungles of Sarawak are considered un-mined treasure-troves of plant species with medicinal properties that are freely available to the animal species living there, including the macaque reservoir of *P*. *knowlesi* [49]. Selection at *Plasmodium* loci, that have evolved to eliminate natural toxins, then assumes biological relevance. Unfortunately these loci also evolve to eliminate antimalarial compounds when used to treat patients with malaria.

*P*. *knowlesi* is a relatively 'un-tamed' *Plasmodium* species, therefore *P*. *knowlesi* genomes may retain ancient and diverse genetic signatures, that are presently invisible in heavily drug selected human-host restricted parasite populations such as *P*. *falciparum* and *P*. *vivax*. High throughput pathogen genome sequencing is a powerful new tool for infectious disease research. Here we use Illumina HiSeq and MiSeq platforms to produce high quality *P*. *knowlesi* genome sequences from difficult archived frozen samples. Analysis of the sequences uncovered a *P*. *knowlesi* genome-wide dimorphism that suggests there are least two types of *P*. *knowlesi* parasites in our patient cohort. We further discovered dimorphic genes among transporter genes that are important in antimalarial drug resistance. Genome-wide pathogen analyses, of even a small number of clinical malaria isolates, instantly added context to our understanding of *Plasmodium* pathobiology, particularly through between-species comparison.

## Supporting Information

S1 Fig *P*. *knowlesi* genome SNP density map. Six *P*. *knowlesi* genome sequences from patient isolates were mapped to the *P*.*knowlesi* reference genome. Sites that differ from the reference are shown as blue bars (all SNP sites) or grey bars (SNP sites co-associating with the *P*. *knowlesi* genome-wide dimorphism). Each bar is 1 pixel wide and represents DNA fragments 809 bases long. The height of the bars represents the number of SNP sites per 809 base fragment. Gaps correspond to regions with low coverage (see results section) or where the reference genome is incomplete (runs of 'N').
(PNG)

S1 Table Distribution of co-associating SNPs by chromosome in six *P*. *knowlesi* genome sequences from human isolates. Each chromosome was divided into 30 equal parts.
(PDF)

S2 Table List of annotated genes from six clinical isolates with >9 SNPs that co—associate with the *Plasmodium knowlesi* genome-wide dimorphism. (PDF)
