# Supplementary figures and images for: Plasmodium knowlesi Genome Sequences from Clinical Isolates Reveal Extensive Genomic Dimorphism
Source: PLoS One. 2015 Apr 1;10(4):e0121303. doi: 10.1371/journal.pone.0121303 (PMC4382175; doi:10.1371/journal.pone.0121303)

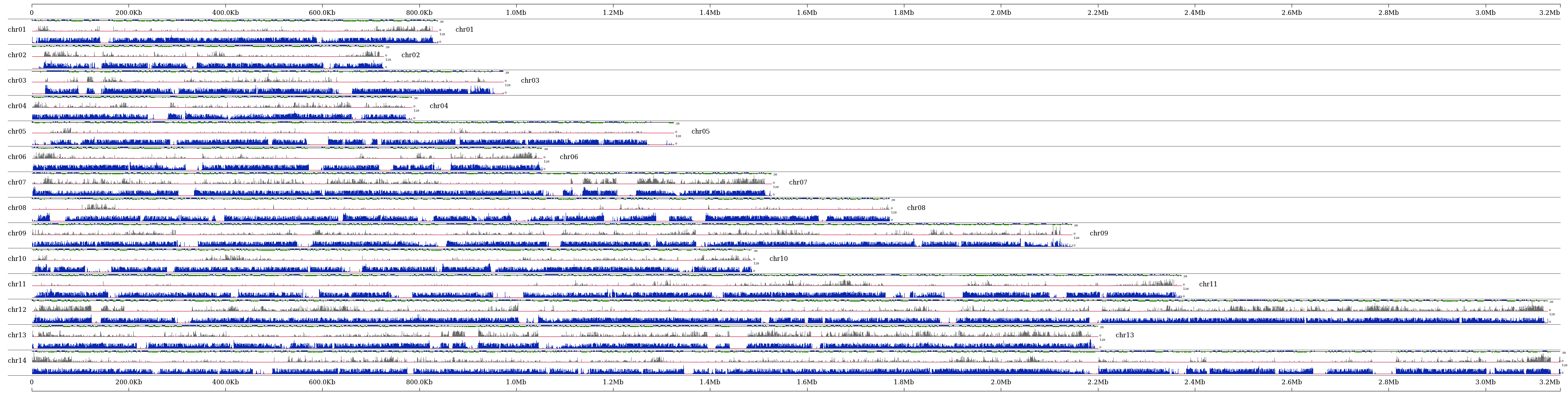

Supplement: S1 Fig — Six P. knowlesi genome sequences from patient isolates were mapped to the P.knowlesi reference genome. Sites that differ from the reference are shown as blue bars (all SNP sites) or grey bars (SNP sites co-associating with the P. knowlesi genome-wide dimorphism). Each bar is 1 pixel wide and represents DNA fragments 809 bases long. The height of the bars represents the number of SNP sites per 809 base fragment. Gaps correspond to regions with low coverage (see results section) or where the reference genome is incomplete (runs of 'N'). (PNG) [file pone.0121303.s001.png]
