# Supplementary material for: Plasmodium knowlesi Genome Sequences from Clinical Isolates Reveal Extensive Genomic Dimorphism
Source: PLoS One. 2015 Apr 1;10(4):e0121303. doi: 10.1371/journal.pone.0121303 (PMC4382175; doi:10.1371/journal.pone.0121303)
Supplement: S1 Table — Each chromosome was divided into 30 equal parts. (PDF) [file pone.0121303.s002.pdf]

**Table S1** Distribution of co-associating SNP's by chromosome in six *P. knowlesi* genome sequences from human isolates.

| Chromosome | Slice 1 | Slice 2 | Slice 3 | Slice 4 | Slice 5 | Slice 6 | Slice 7 | Slice 8 | Slice 9 | Slice 1( | Slice 1 | Slice 1: | Slice 1: | Slice 1 | Slice 1 | Slice 1 | Slice 1 | Slice 1 | Slice 1 | Slice 2( | Slice 2 | Slice 2: | Slice 2: | Slice 2 | Slice 2 | Slice 2 | Slice 2 | Slice 2 | Slice 2 | Slice 3( | Slice size |
|---|---|---|---|---|---|---|---|---|---|---|---|---|---|---|---|---|---|---|---|---|---|---|---|---|---|---|---|---|---|---|---|
| Pk_strainH_chr01 | 4.4 | 0.4 | -4.4 | -3.0 | 3.2 | -4.9 | -1.8 | -2.7 | -2.7 | -2.7 | -2.2 | -2.5 | -4.0 | -0.2 | 0.6 | -1.5 | 1.0 | 3.3 | -1.0 | -4.4 | -4.6 | -6.0 | -4.7 | -2.6 | -2.9 | 5.6 | 9.3 | 15.7 | 5.8 | 1.4 | 27953 |
| Pk_strainH_chr02 | -9.1 | 14.2 | 17.3 | 8.2 | -1.6 | -4.5 | 8.1 | -1.5 | -3.0 | -1.5 | 3.0 | -1.4 | -2.7 | -3.7 | 0.6 | 10.3 | 3.5 | 0.8 | -4.0 | -1.3 | -4.7 | -4.2 | -4.1 | -4.7 | -6.8 | -7.1 | -4.4 | -1.0 | 11.4 | -1.5 | 24229 |
| Pk_strainH_chr03 | -6.6 | -6.8 | 6.1 | 13.7 | 7.5 | 8.8 | -2.3 | -4.7 | -4.8 | -2.2 | 7.4 | 0.3 | 1.6 | 10.1 | 11.1 | 13.2 | 6.5 | 1.2 | 3.1 | -3.3 | -6.5 | -5.1 | -3.4 | -1.4 | -0.9 | -3.4 | -3.8 | -5.4 | -2.2 | -9.9 | 32443 |
| Pk_strainH_chr04 | 3.1 | -2.5 | -2.6 | 0.7 | 2.2 | -3.0 | 3.3 | 9.6 | -2.5 | -5.9 | -0.9 | 1.1 | -0.9 | 1.1 | 1.2 | -2.6 | -2.3 | -0.1 | 0.9 | 2.3 | 3.7 | 2.6 | 4.8 | -1.0 | 10.1 | -5.2 | -2.9 | 0.7 | -2.6 | -8.7 | 26171 |
| Pk_strainH_chr05 | -5.0 | 20.0 | 1.2 | 0.4 | 1.2 | -2.3 | -2.9 | -2.2 | -0.6 | 0.2 | 1.2 | 3.5 | -0.1 | -3.6 | 0.1 | -2.3 | -3.5 | 1.2 | -1.1 | 3.0 | 5.8 | 1.2 | 1.7 | 2.5 | 2.8 | -3.0 | -3.2 | -0.9 | -2.3 | -7.8 | 44166 |
| Pk_strainH_chr06 | 10.4 | 10.4 | -1.4 | -2.0 | -0.2 | -3.8 | -4.3 | -1.6 | -0.1 | -6.8 | 2.5 | 0.3 | 2.2 | -0.9 | -1.7 | -5.3 | -7.1 | -6.4 | -7.7 | 0.6 | -6.6 | -5.9 | 5.2 | -1.8 | -3.9 | -0.7 | 0.4 | 2.6 | 13.7 | 6.3 | 35103 |
| Pk_strainH_chr07 | -2.9 | -4.6 | 7.6 | 5.7 | -0.4 | -3.3 | -8.8 | -0.8 | -1.2 | 4.5 | 6.6 | -4.7 | 8.2 | 11.3 | 4.7 | 4.2 | -3.8 | -8.7 | -11.4 | -9.8 | -12.2 | -9.9 | 6.3 | 8.7 | 1.6 | 15.4 | -5.7 | 0.8 | 7.7 | -2.5 | 50891 |
| Pk_strainH_chr08 | -6.1 | 5.1 | 50.5 | -3.6 | -1.7 | -4.3 | -3.3 | -2.8 | -0.8 | -2.8 | 0.6 | 2.2 | -1.8 | -2.6 | -1.9 | -2.9 | -1.5 | -1.3 | -4.8 | 1.3 | 2.4 | 3.5 | -3.0 | -1.8 | -1.4 | 4.0 | -1.8 | -5.6 | -4.8 | -2.3 | 59011 |
| Pk_strainH_chr09 | -6.6 | -3.1 | 3.4 | 5.6 | -2.6 | -4.3 | -0.9 | 0.9 | 9.0 | 0.9 | -0.3 | -5.3 | 1.0 | 3.5 | 3.4 | 2.3 | 3.6 | -1.3 | 6.6 | 3.3 | 6.7 | 11.2 | 1.6 | -10.3 | -8.2 | -1.4 | -1.7 | 0.6 | -4.9 | -5.2 | 71570 |
| Pk_strainH_chr10 | -0.7 | -5.6 | -6.6 | -4.3 | -2.8 | -5.3 | -4.5 | 11.9 | 28.8 | 7.3 | -2.4 | -3.4 | -4.4 | -4.7 | -2.7 | -3.4 | -5.2 | 0.3 | -4.7 | -5.1 | 2.2 | -0.6 | -1.5 | 3.7 | 3.5 | -0.2 | -0.7 | 1.0 | 2.0 | 4.0 | 49534 |
| Pk_strainH_chr11 | -8.6 | -7.1 | -6.3 | -2.1 | -4.4 | -6.2 | -4.9 | -6.2 | -4.7 | -6.6 | -2.8 | -4.2 | -8.0 | -2.7 | 4.4 | 0.1 | 13.2 | 0.8 | 2.0 | 16.7 | 11.3 | 26.0 | -5.8 | -6.0 | 5.1 | -1.8 | -6.0 | -5.9 | -2.9 | 16.3 | 79096 |
| Pk_strainH_chr12 | 8.4 | 9.5 | -13.7 | 1.3 | 6.3 | 15.0 | 8.1 | -2.7 | -7.5 | 5.6 | -6.4 | -5.7 | -9.7 | -8.5 | -9.6 | -8.7 | -6.6 | 5.7 | -6.5 | -5.9 | -5.6 | -6.8 | 3.8 | 6.3 | 11.0 | 1.3 | 18.8 | -0.1 | -9.4 | 2.4 | 104279 |
| Pk_strainH_chr13 | -6.4 | -8.2 | -5.7 | -9.6 | 3.0 | -6.8 | -7.0 | -9.5 | -6.7 | -0.8 | 3.3 | 13.8 | 10.5 | 4.3 | -4.3 | 2.6 | -0.9 | 4.5 | 6.9 | -7.2 | 14.5 | 3.3 | 1.9 | 9.4 | 1.1 | 2.7 | 0.5 | 3.8 | 2.8 | -9.3 | 73343 |
| Pk_strainH_chr14 | 13.4 | -6.1 | -7.1 | -4.1 | -9.1 | 8.9 | 13.0 | 13.2 | 4.6 | 0.7 | -4.1 | 3.4 | 2.0 | -3.8 | 2.9 | -0.6 | 5.4 | -2.4 | 13.0 | 13.9 | -6.3 | -5.0 | -8.1 | -9.9 | -12.8 | -10.4 | -9.3 | -4.9 | -3.9 | 11.0 | 105119 |

Green - distribution of co-associating SNP's less than expected
Red - distribution of co-associating SNP's more than expected
Each chromosome divided into 30 fragments, 'slices'.
