# Supplementary material for: Plasmodium knowlesi Genome Sequences from Clinical Isolates Reveal Extensive Genomic Dimorphism
Source: PLoS One. 2015 Apr 1;10(4):e0121303. doi: 10.1371/journal.pone.0121303 (PMC4382175; doi:10.1371/journal.pone.0121303)
Supplement: S2 Table — (PDF) [file pone.0121303.s003.pdf]

Table S2 List of annotated genes from six clinical isolates with >9 SNP's that co - associate with the *Plasmodium knowlesi* genome-wide dimorphism.

Gene ID	Gene function	No Co-assoc SNPs	Total SNPs	% co-assoc SNPs
PKH_010260	phosphoinositide-binding protein, putative	11	68	16.2
PKH_010740	Cg2-like protein	12	259	4.6
PKH_011630	transcription factor with AP2 domain(s), putative (ApiAP2)	34	230	14.8
PKH_011640	gluatamate dehydrogenase, putative (GDH3)	13	60	21.7
PKH_011650	reductase, putative	76	339	22.4
PKH_011660	mannose-6-phosphate isomerase, putative	12	77	15.6
PKH_011670	sentrin-specific protease 2, putative (SENP2)	28	180	15.6
PKH_011700	protein kinase, putative	15	76	19.7
PKH_011714	unspecified product	10	80	12.5
PKH_020300	phosphatidylinositol-4-phosphate 5-kinase, putative (PIP5K)	15	141	10.6
PKH_020580	patched family protein, putative	13	79	16.5
PKH_020750	asparagine-rich antigen, putative	14	213	6.6
PKH_021090	ubiquitin carboxyl-terminal hydrolase, putative	10	224	4.5
PKH_030250	RNA binding protein, putative	13	96	13.5
PKH_030610	sporozoite invasion-associated protein 1, putative (SIAP1)	15	56	26.8
PKH_030890	lysine decarboxylase, putative	17	165	10.3
PKH_030920	pre-mRNA-processing-splicing factor 8, putative (PRPF8)	10	199	5
PKH_031060	transcription factor with AP2 domain(s), putative (ApiAP2)	27	403	6.7
PKH_040430	DNA-directed RNA polymerase II second largest subunit, putative	11	74	14.9
PKH_040490	ATP-dependent acyl-CoA synthetase, putative	16	42	38.1
PKH_041100	6-cysteine protein (P230)	27	175	15.4
PKH_041110	6-cysteine protein (P230p)	18	134	13.4
PKH_041220	cysteine protease, putative	14	89	15.7
PKH_041240	cysteine protease, putative	11	79	13.9
PKH_041310	merozoite surface protein 5	10	35	28.6
PKH_041490	DEAD/DEAH box helicase, putative	10	33	30.3
PKH_041520	5'-3' exonuclease, putative	25	83	30.1
PKH_041680	protein kinase, putative	30	205	14.6
PKH_050090	tryptophan/threonine-rich antigen, putative	14	23	60.9
PKH_050110	sporozoite invasion-associated protein 2, putative (SIAP-2)	12	34	35.3
PKH_051180	MAC/perforin, putative	10	48	20.8
PKH_052430	phosphatidylinositol 4-kinase, putative	23	351	6.6
PKH_060230	erythrocyte membrane-associated antigen, putative	14	198	7.1
PKH_060310	eukaryotic translation initiation factor subunit eIF2A, putative	10	207	4.8
PKH_060730	dynein heavy chain, putative	15	342	4.4
PKH_060810	chromodomain-helicase-DNA-binding protein 1 homolog, putative (CHD1)	16	201	8
PKH_061750	S-adenosylmethionine decarboxylase-ornithine decarboxylase, putative	22	84	26.2
PKH_061820	aspartyl protease, putative	10	64	15.6
PKH_062240	GDP dissociation inhibitor, putative	24	94	25.5
PKH_062280	KIR-like protein	14	24	58.3
PKH_070180	ubiquitin specific protease, putative	29	139	20.9
PKH_070210	E1-E2_ATPase/hydrolase, putative	40	186	21.5
PKH_070230	nucleoporin NUP100/NSP100, putative (NUP100)	18	103	17.5
PKH_070250	dynein heavy chain, putative	35	311	11.3
PKH_070260	rhoptry protein, putative	14	41	34.1
PKH_070680	exoribonuclease, putative	11	109	10.1
PKH_070750	Ran-binding protein, putative	21	75	28
PKH_070790	DNA repair protein REV1, putative	11	121	9.1
PKH_071250	ribonuclease H2 subunit C, putative	10	43	23.3
PKH_071270	6-phosphofructokinase, putative (PFK9)	17	77	22.1
PKH_071330	transporter, putative	21	123	17.1
PKH_071660	protein disulfide-isomerase, putative	10	35	28.6
PKH_071710	regulator of chromosome condensation, putative	27	284	9.5
PKH_072500	zinc finger protein, putative	22	108	20.4
PKH_072660	serine/threonine protein kinase, putative	13	41	31.7
PKH_072670	guanylate kinase, putative (GK)	10	17	58.8
PKH_072830	leucine-rich repeat protein (LRR8)	20	48	41.7
PKH_072870	diacylglycerol kinase, putative	10	84	11.9
PKH_072930	GTP binding protein, putative	12	65	18.5
PKH_073170	mitochondrial-processing peptidase subunit beta, putative (MAS1)	12	20	60
PKH_073220	DNA excision-repair helicase, putative	16	97	16.5
PKH_073320	vesicle transport protein, putative	31	123	25.2
PKH_073340	gametocyte development protein 1, putative (GDV1)	21	50	42
PKH_073430	lysophospholipase, putative	19	90	21.1
PKH_080250	U5 small nuclear ribonuclear protein, putative	33	81	40.7
PKH_083000	phd finger protein, putative	11	448	2.5
PKH_090100	CCR4-NOT transcription complex subunit 1, putative (NOT1)	12	256	4.7
PKH_090140	chromatin remodeling protein, putative (SNF2L)	11	82	13.4
PKH_090460	polyadenylate-binding protein-interacting protein 1, putative (PAIP1)	32	246	13
PKH_090510	transcription factor with AP2 domain(s), putative (ApiAP2)	21	145	14.5
PKH_091340	rhoptry neck protein 4, putative (RON4)	15	97	15.5
PKH_091350	serine esterase, putative	33	131	25.2
PKH_091410	cathepsin C precursor, putative	12	32	37.5
PKH_092060	asparagine-rich protein, putative	11	152	7.2
PKH_092110	RAP protein, putative	11	83	13.3
PKH_092160	beige/BEACH domain protein, putative	13	119	10.9
PKH_092360	threonine--tRNA ligase, putative (ThrRS)	16	63	25.4
PKH_092450	RNA binding protein, putative	17	103	16.5
PKH_092690	parasitophorous vacuolar protein 1, putative (PV1)	10	33	30.3
PKH_092760	transporter, putative	10	25	40
PKH_093150	RNA (uracil-5-)methyltransferase, putative	17	71	23.9
PKH_093250	zinc finger protein, putative	25	197	12.7
PKH_093290	protein phosphatase, putative	17	104	16.3
PKH_093420	tudor staphylococcal nuclease, putative (TSN)	11	63	17.5
PKH_093620	guanylyl cyclase, putative	31	241	12.9

PKH_094060	transcription factor with AP2 domain(s), putative (AP2-O)	19	147	12.9
PKH_094280	protein kinase, putative	11	157	7
PKH_094440	sporozoite asparagine-rich protein (SLARP)	31	272	11.4
PKH_094520	protein kinase, putative	11	49	22.4
PKH_100020	KIR-like protein	15	123	12.2
PKH_100820	karyopherin beta, putative	16	48	33.3
PKH_100840	transporter, putative	12	34	35.3
PKH_103010	merozoite surface protein 3b, putative	12	73	16.4
PKH_103190	rhoptry-associated protein 3, putative	15	34	44.1
PKH_111420	serine/threonine protein kinase, putative (ARK3)	10	435	2.3
PKH_112660	transcription factor with AP2 domain(s), putative (ApiAP2)	22	273	8.1
PKH_113250	TRAP-like protein, putative (TLP)	36	132	27.3
PKH_113520	transcription factor with AP2 domain(s), putative (ApiAP2)	118	462	25.5
PKH_113530	syntaxin binding protein, putative	23	64	35.9
PKH_113660	leucine-rich repeat protein (LRR6)	16	140	11.4
PKH_114550	transcription factor with AP2 domain(s), putative,SPE2-interacting protein, putative (SIP2	10	213	4.7
PKH_114780	ATP-dependant RNA helicase, putative	10	78	12.8
PKH_120090	tryptophan-rich antigen	14	32	43.8
PKH_120110	vacuolar fusion protein MON1, putative	12	38	31.6
PKH_120190	ABC transporter, putative	21	64	32.8
PKH_120230	alveolin, putative (ALV6)	32	134	23.9
PKH_120290	phosphatidylinositol transfer protein, putative	43	103	41.7
PKH_120350	ubiquitin-activating enzyme e1, putative	13	80	16.2
PKH_120460	serine/threonine protein kinase, putative (TKL3)	41	134	30.6
PKH_121160	transcription factor with AP2 domain(s), putative (ApiAP2)	45	381	11.8
PKH_121190	myosin A, putative	10	25	40
PKH_121210	Casein kinase II regulatory subunit, putative	10	38	26.3
PKH_121330	sodium-dependent phosphate transporter, putative (PiT)	10	40	25
PKH_121390	nucleolar complex protein 2, putative	18	55	32.7
PKH_121420	ABC transporter, putative	15	67	22.4
PKH_121770	sporozoite surface protein 2, putative,thrombospondin-related anonymous protein (TRA	18	38	47.4
PKH_121970	ubiquitin activating enzyme, putative (UBA1)	10	132	7.6
PKH_122360	myosin, putative	16	144	11.1
PKH_122410	alpha/beta-hydrolase, putative	27	75	36
PKH_123100	SET domain protein, putative	25	262	9.5
PKH_123980	dynein beta chain, putative	18	333	5.4
PKH_124700	GTP-binding protein, putative	11	60	18.3
PKH_125210	aminopeptidase P, putative (APP)	11	38	28.9
PKH_125270	glucose-6-phosphate dehydrogenase-6-phosphogluconolactonase, putative	11	43	25.6
PKH_125430	rhoptry neck protein 2, putative (RON2)	60	156	38.5
PKH_125490	transcriptional regulatory protein sir2b, putative (Sir2b)	13	91	14.3
PKH_125590	zinc finger protein, putative	13	129	10.1
PKH_125660	exosome complex exonuclease RRP6, putative (RRP6)	12	121	9.9
PKH_125680	transcription factor with AP2 domain(s), putative (ApiAP2)	11	95	11.6
PKH_125830	TRAP-like protein (TREP)	25	86	29.1
PKH_125840	multidrug resistance protein 2, putative	48	133	36.1
PKH_125950	Glutamine cyclotransferase, putative	11	25	44
PKH_126090	serine/threonine kinase-1, putative	12	45	26.7
PKH_126180	Ser/Thr protein kinase, putative	15	102	14.7
PKH_126240	heat shock protein, putative	13	64	20.3
PKH_126270	spindle pole body protein, putative	37	117	31.6
PKH_126280	Poly(A)-specific ribonuclease PARN, putative (PARN)	22	72	30.6
PKH_126340	guanine-nucleotide-exchange-factor, putative	25	192	13
PKH_126380	protein prenyltransferase alpha subunit, putative	22	56	39.3
PKH_126410	probable GTP-binding protein, putative	31	78	39.7
PKH_126490	FACT complex subunit SSRP1, putative (FACT-S)	10	28	35.7
PKH_126500	serine/threonine protein kinase, putative	63	254	24.8
PKH_126680	Histone-like transcription factor, putative	23	150	15.3
PKH_126880	protein kinase, putative	11	66	16.7
PKH_126950	cysteine repeat modular protein 4, putative (CRMP4)	82	340	24.1
PKH_126970	bromodomain containing protein, putative	11	48	22.9
PKH_130070	ATP-dependent RNA helicase, putative	15	49	30.6
PKH_130610	small subunit rRNA processing factor, putative	10	74	13.5
PKH_130710	cysteine repeat modular protein 3, putative (CRMP3)	48	252	19
PKH_130840	3',5'-cyclic-nucleotide phosphodiesterase, putative	13	71	18.3
PKH_131400	NAD(P)H-dependent glutamate synthase, putative	29	189	15.3
PKH_131480	P-loop containing nucleoside triphospahte hydrolase, putative	42	661	6.4
PKH_131540	protein kinase, putative	26	219	11.9
PKH_131690	leucine-rich repeat protein (LRR5)	15	171	8.8
PKH_131750	knob-associated histidine-rich protein, putative (KAHRP)	19	52	36.5
PKH_131840	syntaxin, putative	21	59	35.6
PKH_132040	ATP-dependent DNA helicase, putative	14	66	21.2
PKH_132090	rRNA methylase, putative	28	218	12.8
PKH_132240	WW domain-binding protein 11, putative	15	48	31.2
PKH_132330	lipase, putative	20	99	20.2
PKH_132370	phosphoenolpyruvate carboxylase, putative (PEPC)	17	62	27.4
PKH_132390	ATP-binding cassette sub-family G member 2, putative (ABCG2)	11	43	25.6
PKH_132990	ATP-dependent RNA helicase, putative	12	53	22.6
PKH_133050	cysteine protease ATG4, putative (ATG4)	29	102	28.4
PKH_133100	metacaspase-like protein (MCA3)	37	204	18.1
PKH_133110	GTP binding protein, putative	12	52	23.1
PKH_133220	uracil-DNA glycosylase, putative	11	27	40.7
PKH_133250	ubiquitin carboxyl-terminal hydrolase, putative	11	112	9.8
PKH_133270	atypical protein kinase, ABC-1 family, putative	44	152	28.9
PKH_133490	ATPase, putative	31	95	32.6
PKH_133600	GTP binding protein, putative	15	41	36.6
PKH_133620	POM1 homolog, putative (PREX)	19	128	14.8
PKH_133730	rhoptry-associated protein 1, putative (RAP1)	17	52	32.7
PKH_133780	cytidine diphosphate-diacylglycerol synthase (CDS)	21	32	65.6

PKH_133850	oxidoreductase, aldo/keto reductase domain containing protein	11	83	13.3
PKH_133910	transcription factor with AP2 domain(s), putative (ApiAP2)	44	123	35.8
PKH_134030	ATP-dependent Clp protease, putative	14	88	15.9
PKH_134120	circumsporozoite protein, putative	13	60	21.7
PKH_134140	COBW domain-containing protein 1, putative (CBWD1)	10	32	31.2
PKH_134230	adenylyl cyclase 1, putative	17	55	30.9
PKH_134310	nuclear formin-like protein (MISFIT)	44	167	26.3
PKH_134580	erythrocyte binding protein (gamma)	17	86	19.8
PKH_140230	peptidase, putative	59	741	8
PKH_140780	mRNA-decapping enzyme 2, putative (DCP2)	12	90	13.3
PKH_141200	eukaryotic translation initation factor 4 gamma, putative (EIF4G)	21	77	27.3
PKH_141520	Exportin-1 domain containig protein	10	69	14.5
PKH_141540	inositol polyphosphate kinase, putative (IPK2)	16	145	11
PKH_141620	U4/U6.U5 tri-snRNP-associated protein 2, putative (USP39)	13	48	27.1
PKH_142160	zinc finger protein, putative	13	77	16.9
PKH_142730	CCR4-associated factor 1, putative (CAF1)	19	164	11.6
PKH_142840	ABC transporter, putative	15	62	24.2
PKH_142870	JmjC domain containing protein	24	51	47.1
PKH_143610	formin 2, putative	15	245	6.1
PKH_143910	transcription factor with AP2 domain(s), putative (ApiAP2)	18	232	7.8
PKH_144190	isoleucine--tRNA ligase, putative	10	161	6.2
PKH_144230	ribosome biogenesis protein, putative	10	38	26.3
PKH_144260	ubiquitin-activating enzyme e1, putative	21	58	36.2
PKH_144590	multidrug resistance-associated protein 1, putative (MRP1)	89	198	44.9
PKH_144890	asparagine-rich protein, putative	18	193	9.3
PKH_145070	asparagine/aspartate rich protein, putative	16	442	3.6
PKH_145310	transcription factor with AP2 domain(s), putative (ApiAP2)	14	322	4.3
PKH_146780	pfg377 homolog, putative	27	162	16.7
PKH_146850	DNA repair protein rhp16, putative	12	101	11.9
PKH_146940	tryptophan--tRNA ligase, putative	11	49	22.4
PKH_146970	reticulocyte binding protein, putative (fragment)	91	326	27.9
PKH_146990	tryptophan-rich antigen	27	70	38.6
